# Performance of the GeneXpert Ebola Assay for Diagnosis of Ebola Virus Disease in Sierra Leone: A Field Evaluation Study

**DOI:** 10.1371/journal.pmed.1001980

**Published:** 2016-03-29

**Authors:** Amanda E. Semper, M. Jana Broadhurst, Jade Richards, Geraldine M. Foster, Andrew J. H. Simpson, Christopher H. Logue, J. Daniel Kelly, Ann Miller, Tim J. G. Brooks, Megan Murray, Nira R. Pollock

**Affiliations:** 1 Public Health England, Porton Down, United Kingdom; 2 Partners In Health, Boston, Massachusetts, United States of America; 3 Public Health England Laboratory, Port Loko, Sierra Leone; 4 Mid Essex Hospital Services NHS Trust, Chelmsford, United Kingdom; 5 Liverpool School of Tropical Medicine, Liverpool, United Kingdom; 6 Wellbody Alliance, Freetown, Sierra Leone; 7 Harvard Medical School, Boston, Massachusetts, United States of America; 8 Department of Laboratory Medicine, Boston Children’s Hospital, Boston, Massachusetts, United States of America; Mahidol-Oxford Tropical Medicine Research Unit, THAILAND

## Abstract

**Background:**

Throughout the Ebola virus disease (EVD) epidemic in West Africa, field laboratory testing for EVD has relied on complex, multi-step real-time reverse transcription PCR (RT-PCR) assays; an accurate sample-to-answer RT-PCR test would reduce time to results and potentially increase access to testing. We evaluated the performance of the Cepheid GeneXpert Ebola assay on clinical venipuncture whole blood (WB) and buccal swab (BS) specimens submitted to a field biocontainment laboratory in Sierra Leone for routine EVD testing by RT-PCR (“Trombley assay”).

**Methods and Findings:**

This study was conducted in the Public Health England EVD diagnostic laboratory in Port Loko, Sierra Leone, using residual diagnostic specimens remaining after clinical testing. EDTA-WB specimens (*n* = 218) were collected from suspected or confirmed EVD patients between April 1 and July 20, 2015. BS specimens (*n* = 71) were collected as part of a national postmortem screening program between March 7 and July 20, 2015. EDTA-WB and BS specimens were tested with Xpert (targets: glycoprotein [GP] and nucleoprotein [NP] genes) and Trombley (target: NP gene) assays in parallel. All WB specimens were fresh; 84/218 were tested in duplicate on Xpert to compare WB sampling methods (pipette versus swab); 43/71 BS specimens had been previously frozen.

In all, 7/218 (3.2%) WB and 7/71 (9.9%) BS samples had Xpert results that were reported as “invalid” or “error” and were excluded, leaving 211 WB and 64 BS samples with valid Trombley and Xpert results. For WB, 22/22 Trombley-positive samples were Xpert-positive (sensitivity 100%, 95% CI 84.6%–100%), and 181/189 Trombley-negative samples were Xpert-negative (specificity 95.8%, 95% confidence interval (CI) 91.8%–98.2%). Seven of the eight Trombley-negative, Xpert-positive (Xpert cycle threshold [Ct] range 37.7–43.4) WB samples were confirmed to be follow-up submissions from previously Trombley-positive EVD patients, suggesting a revised Xpert specificity of 99.5% (95% CI 97.0%–100%). For Xpert-positive WB samples (*n* = 22), Xpert NP Ct values were consistently lower than GP Ct values (mean difference −4.06, 95% limits of agreement −6.09, −2.03); Trombley (NP) Ct values closely matched Xpert NP Ct values (mean difference −0.04, 95% limits of agreement −2.93, 2.84). Xpert results (positive/negative) for WB sampled by pipette versus swab were concordant for 78/79 (98.7%) WB samples, with comparable Ct values for positive results. For BS specimens, 20/20 Trombley-positive samples were Xpert-positive (sensitivity 100%, 95% CI 83.2%–100%), and 44/44 Trombley-negative samples were Xpert-negative (specificity 100%, 95% CI 92.0%–100%). This study was limited to testing residual diagnostic samples, some of which had been frozen before use; it was not possible to test the performance of the Xpert Ebola assay at point of care.

**Conclusions:**

The Xpert Ebola assay had excellent performance compared to an established RT-PCR benchmark on WB and BS samples in a field laboratory setting. Future studies should evaluate feasibility and performance outside of a biocontainment laboratory setting to facilitate expanded access to testing.

## Abstract 2

Nira Pollock and colleagues evaluate the performance of the GeneXpert Ebola assay for diagnosis of clinical and post-mortem specimens submitted to a field laboratory in Sierra Leone.

## Editors' Summary

**Background:**

During the recent Ebola virus disease (EVD) outbreak in West Africa, there were more than 28,000 confirmed, probable, and suspected cases of EVD and more than 11,000 deaths from EVD. Ebola virus is transmitted to people from wild animals and spreads in human populations through contact with the bodily fluids (including blood, saliva, and urine) or organs of infected people and through contact with bedding and other materials contaminated with bodily fluids. The symptoms of EVD, which start 2–21 days after infection, include fever, headache, vomiting, diarrhea, and internal and external bleeding. Infected individuals are not infectious until they develop symptoms, but they remain infectious as long as their bodily fluids contain virus, which can be several weeks. There is no proven treatment for EVD, but supportive care—given under strict isolation conditions to prevent the spread of the disease—improves survival. Currently, there are no licensed Ebola vaccines, but two candidate vaccines are being evaluated.

**Why Was This Study Done?:**

EVD diagnosis during the recent epidemic relied on multi-step reverse transcription polymerase chain reaction (RT-PCR) assays (for example, the Trombley assay) performed in field biocontainment laboratories on blood obtained from a vein using a needle (venipuncture) or on samples of cells and saliva collected from the mouth lining using a swab (buccal swab); buccal swabs are mainly used for surveillance, particularly postmortem screening. Prior to RT-PCR, the sample must be inactivated and nucleic acid extracted. An accurate, fully automated “sample-to-answer” assay that is capable of testing both whole blood and buccal swabs and that minimizes the potential exposure of laboratory personnel to the Ebola virus could greatly improve EVD diagnosis. Here, the researchers evaluate the performance of the Cepheid GeneXpert Ebola assay on whole blood samples and buccal swabs sent to a field laboratory in Sierra Leone for routine testing using the Trombley assay. The Xpert assay is an automated RT-PCR system that integrates all the steps needed to determine the presence of Ebola virus; once the test sample has been inactivated and added to a proprietary cartridge, no further operator action is necessary to generate the result.

**What Did the Researchers Do and Find?:**

The researchers tested 218 whole blood specimens collected from patients with suspected or confirmed EVD using both the Trombley and the Xpert assay. After excluding a few samples that gave Xpert results that were reported as “invalid” or “error,” 22 out of 22 Trombley-positive samples were Xpert-positive, and 181 out of 189 Trombley-negative samples were Xpert-negative. That is, the Xpert assay had a sensitivity (true positive rate) of 100% and a specificity (true negative rate) of 95.8% compared to the benchmark assay. Notably, seven of the eight Trombley-negative but Xpert-positive blood samples were follow-up samples obtained from previously Trombley-positive patients, which suggests that the specificity of the Xpert test was actually 99.5%. When the Xpert assay was used to test 71 buccal swabs, the sensitivity and specificity of the Xpert assay were both 100%. Finally, Xpert results obtained using a pipette versus a swab to pick up a portion of blood for testing were concordant in 78 out of 79 samples; this test was done in part to get an indication of whether the Xpert Ebola assay will work on fingerstick samples—blood samples obtained by using a sterile lancet to pierce the fingertip and then collecting the blood with a swab; this collection method is more easily done in the field at point of care than venipuncture.

**What Do These Findings Mean?:**

These findings show that, compared to an established RT-PCR Ebola virus assay, the Xpert Ebola assay performed well on both whole blood samples and buccal swabs in a field laboratory setting. Although sampling of blood with a swab partly simulated the performance of the Xpert assay on fingerstick samples collected at point of care, fingerstick sample collection will need to be tested directly before the Xpert assay can be used to test individuals for Ebola virus by this method at point of care. Further studies are also needed to evaluate the feasibility and performance of the Xpert assay in a range of clinical settings to determine where and when this assay can be deployed; the need for an uninterrupted power supply and, in some settings, for refrigeration of reagents may prevent its deployment in some resource-limited settings. Ultimately though, these findings suggest that the use of the Xpert Ebola assay could facilitate expanded access to Ebola virus testing.

**Additional Information:**

This list of resources contains links that can be accessed when viewing the PDF on a device or via the online version of the article at http://dx.doi.org/10.1371/journal.pmed.1001980.

- The World Health Organization (WHO) provides information about EVD, information about potential EVD vaccines and therapies, and regular updates on the West African EVD epidemic; the WHO website also provides information about efforts to control Ebola in the field and personal stories from people who have survived EVD
- The UK National Health Service Choices website provides detailed information on EVD
- The US Centers for Disease Control and Prevention also provides information about EVD
- More information on the Xpert Ebola test and authorization for its emergency use is available from the US Food and Drug Administration and from WHO

## Introduction

The prolonged and devastating outbreak of Ebola virus disease (EVD) in West Africa has exposed a need for improved diagnostic methods to reduce result turnaround times and increase access to testing without sacrificing test accuracy. To date, diagnosis of EVD has relied primarily on real-time reverse transcription PCR (RT-PCR) performed in field biocontainment laboratories on submitted venous whole blood (WB) or buccal swab (BS) specimens. Conventional RT-PCR-based testing is cumbersome, requiring multiple processing steps from sample lysis through nucleic acid extraction to the RT-PCR step itself. The use of blood samples has created both logistical complications and biosafety concerns due to requirements for (a) collection and transport of venipuncture blood (often collected with inadequate precautions due to resource limitations, and then transported over substantial distances) and (b) multi-step processing of this blood by skilled operators in a laboratory with adequate biocontainment capacity. Buccal swabs have been used occasionally as diagnostic samples during the outbreak and are being used extensively as part of the ongoing surveillance effort, particularly for postmortem screening. A highly accurate and fully automated RT-PCR assay capable of testing both blood and BS specimens could greatly improve diagnostic capacity in countries affected by the outbreak.

The Cepheid GeneXpert Ebola assay received US Food and Drug Administration emergency use authorization in March 2015 and WHO approval for emergency procurement in May 2015; CE in vitro diagnostic compliance status was confirmed August 10, 2015. The GeneXpert platform is an automated “sample-to-answer” system, integrating sample preparation, virus inactivation, nucleic acid amplification, and detection; once inactivated sample is added to a proprietary cartridge and loaded onto the platform, no further operator action is necessary to generate the result. The system has already demonstrated feasibility for use in resource-limited settings for diagnosis of tuberculosis (e.g., [1]) and, accordingly, has been widely deployed. The Xpert Ebola assay is a two-target RT-PCR assay separately detecting the glycoprotein (GP) and nucleoprotein (NP) genes of the Ebola virus (EBOV). The Ebola assay also includes two internal controls: an exogenous sample processing control (SPC) to determine whether amplification is inhibited during testing, and a sample adequacy control (SAC) that detects an endogenous human genomic DNA target (human hydroxymethylbilane synthase gene) [2] to confirm that sufficient host cellular material is present and intact in the sample. Xpert Ebola assay components (buffers and cartridges) are optimally refrigerated (although temperatures up to 28°C are tolerated), and the GeneXpert platform requires an uninterrupted electricity supply. In this study, we sought to evaluate the field performance of the Xpert Ebola assay on WB and BS samples submitted to an established field biocontainment laboratory in Sierra Leone for routine clinical testing by a well-established RT-PCR benchmark (Trombley assay) [3,4].

## Methods

### Ethics Statement

The study protocol was approved by the Sierra Leone Ethics and Scientific Review Committee, the Sierra Leone Pharmacy Board, and the Partners Human Research Committee (Boston, MA, US). Testing of excess specimens submitted for routine clinical testing was approved with a waiver of informed consent.

### Study Design and Setting

We conducted this study in the Public Health England (PHE) EVD diagnostic laboratory in Port Loko, Sierra Leone (henceforth referred to as the field laboratory [FL]). Fresh EDTA-WB venipuncture specimens (*n* = 218) submitted for routine clinical EVD diagnostic testing between April 1 and July 20, 2015, first underwent clinical testing with the laboratory’s standard RT-PCR procedure (Trombley assay) and then were evaluated using the Xpert Ebola assay on the GeneXpert platform. Given the automated sample-to-answer design of the Xpert Ebola assay, it was not considered necessary to blind operators performing the Xpert assay to the results of the Trombley assay. Fresh BS specimens (*n* = 28) submitted for clinical RT-PCR testing (Trombley assay) between April 5 and July 20, 2015, as well as a selected set of frozen BS specimens (*n* = 43, collected between March 7 and May 1, 2015) were also evaluated by the Xpert Ebola assay; for previously frozen swabs, the Trombley assay was repeated in parallel with Xpert testing. Initial plans for sample testing were to test all WB and BS samples consecutively submitted to the laboratory. However, because of decreasing case rates over the course of the study, we ultimately selected some samples for testing by Xpert based on prior Trombley results (positive/negative). In sum, approximately 50 laboratory staff members participated in testing using the Trombley assay, Xpert assay, or both. All staff were trained utilizing a formal training procedure before operating instruments and interpreting tests.

### Clinical RT-PCR Testing Procedures (Trombley Assay)

On receipt in the FL, EDTA-WB samples and BS samples collected and transported in Sigma-Virocult tubes (Medical Wire and Equipment) were transferred to a flexible film isolator (FFI) for lysis and inactivation. For blood samples, viral RNA was typically manually extracted from 50 μl of plasma using the QIAamp Viral RNA Kit (Qiagen); sample extraction volumes were adjusted from those recommended by Qiagen to minimize inhibition observed when larger volumes of field samples were used. For ~15% of blood samples, 80 μl of plasma was extracted using the EZ1 Virus Mini Kit v2.0 (Qiagen) in conjunction with the EZ1 platform (Qiagen).

For fresh and frozen BS specimens, the swab was agitated in the viral transport medium, and the medium then harvested and centrifuged at 3,000 rpm (665*g*) for 1–2 min. RNA was extracted from 25 μl of clarified supernatant using the EZ1 Virus Mini Kit v2.0 (Qiagen) in conjunction with the EZ1 platform (Qiagen); for four fresh BS samples, RNA was extracted manually using the QIAamp Viral RNA Kit (Qiagen).

Intact MS2 phage was included in all RNA extractions as an exogenous internal control. Extracts were resuspended in 60 μl of AVE buffer (Qiagen), and qualitative RT-PCR for detection of EBOV was performed using the Trombley assay [3,4]. Samples were usually tested on the day of receipt in the laboratory; samples received in the evening were tested the next morning (maximum delay of 12 h). Details of Trombley assay performance have been described recently [4]. Duplex RT-PCR was performed with primers/probes directed against the EBOV NP gene (FAM channel) and the MS2 genome (Alx532 channel; in-house assay) using TaqMan Fast Virus 1-Step Master Mix (Applied Biosystems) on a SmartCycler II platform (Cepheid). Assays were conducted with the following cycling conditions: 50°C for 5 min (one cycle); 95°C for 20 s (one cycle); 95°C for 3 s and 60°C for 30 s (45 cycles). A single fluorescence read was taken at the end of each 60°C step. Samples with cycle threshold (Ct) ≥ 40 and a positive internal control were interpreted as EBOV negative; if the internal control failed, the result was interpreted as a sample failure, and the test was repeated. Samples with Ct > 0 and < 40 with or without a positive internal control were interpreted as EBOV positive.

### Xpert Ebola Assay Testing Procedures

The GeneXpert Ebola assay was performed using the GeneXpert Ebola Assay kit (REBOLA-50; Cepheid), as per the manufacturer’s instructions (RUO package insert 301–4732, Revision 1, March 2015). All steps prior to loading test cartridges onto the Xpert instrument platform were carried out in FFIs. EDTA-WB was prepared either by pipetting 100 μl of blood into 2.5 ml of sample reagent buffer or by allowing EDTA-WB to wick up one of the swabs provided with the Xpert Ebola test kits until the swab was fully wetted, then transferring the swab to 2.5 ml of sample reagent buffer (both sampling methods are recommended by the manufacturer). Fresh BS specimens were agitated in their Sigma-Virocult tube, and 100 μl of crude swab medium was then transferred to 2.5 ml of sample reagent buffer. Fresh excess clinical samples were usually tested by Xpert within 1–2 d of testing by Trombley assay, but storage of either the fresh clinical sample or the sample in Xpert sample reagent buffer at 4°C for up to 6 d prior to testing on Xpert was permitted. Archived BS samples, stored at −80°C as crude BS medium, were thawed (either for 30 min at room temperature or for 24 h at 4°C), then gently agitated before transferring 100 μl of medium to 2.5 ml of sample buffer.

All samples were incubated in sample reagent for 20 min at room temperature, then transferred to Xpert Ebola test cartridges, taking care not to introduce air bubbles. Cartridges were loaded onto a GeneXpert XVI platform (Cepheid), operated according to the manufacturer’s instructions. The following results were interpreted as Ebola positive: Ebola GP detected and Ebola NP detected; Ebola GP detected and Ebola NP not detected; Ebola GP not detected and Ebola NP detected. Results were interpreted as Ebola negative if neither Ebola GP nor Ebola NP was detected, providing the SPC and SAC controls passed. Negative tests in which the SPC or SAC failed or fell outside a cutoff Ct value preset by the manufacturer were reported by the system software as “invalid.” Tests were reported by the system as “error” if the probe check control failed or process errors occurred (e.g., “motion of syringe drive not detected”). Tests in which inadequate data were collected by the system (e.g., the test run was interrupted due to sample loading error or power loss during the test run) were reported by the system software as “no result.” When possible, tests reported as invalid/error/no result were repeated using newly extracted sample. If a valid result was obtained on repeat testing, the valid result was used for analysis.

### Data Management and Statistical Analysis

Data entry was independently performed by at least two different members of the study team, and the data entered were then reconciled. Sensitivity was defined for each specimen type as the proportion of Trombley RT-PCR-positive samples that tested positive by the Xpert Ebola assay, and specificity was defined for each specimen type as the proportion of Trombley RT-PCR-negative samples that tested negative by the Xpert Ebola assay. Samples that generated a final report of invalid/error/no result from the Xpert Ebola assay were excluded from sensitivity and specificity calculations. 95% CIs for proportions were calculated using the exact binomial (Clopper—Pearson) formula. Method agreement was assessed by Bland—Altman analysis [5] using Stata 13 (StataCorp).

This study has been reported according to STARD guidelines (S1 Table).

## Results

### Testing of Residual Clinical EDTA—Whole Blood Specimens

Patient gender was recorded for 186/218 WB specimens, of which 109 (58.6%) were from male and 77 (41.4%) from female patients. Patient age was recorded for 192 specimens, and ranged from 11 mo to 105 y (mean 33.2 y, standard deviation [SD] 17.4; median 32, interquartile range [IQR] 22–41). In all, 94 (43.3%) of the WB specimens were tested by Xpert and Trombley assays on the same day. The remaining specimens were tested by the Xpert assay 1–6 d following Trombley testing (mean 1.8 d, SD 1.1; median 1, IQR 1–2). Ambient temperatures in the FL during testing procedures ranged from 23.0 to 31.7°C (mean 27.9), and ambient humidity ranged from 45% to 87% (mean 64.9%).

Nine of 218 WB specimens initially generated an Xpert report of “invalid,” “error,” or “no result.” Four such results were due to internal control failures (two SPC failures, one SAC failure, and one sample with both SPC and SAC failures; all reported as “invalid”), three were due to probe check or process errors (reported as “error”), and two were due to automated loading errors during the assay run (reported as “no result”) (detailed results are provided in S2 Table). Adequate volume was available for repeat testing of two of these specimens (the two associated with loading errors on initial testing), both of which generated valid results that were included in subsequent analyses per the study protocol. The seven specimens with insufficient volume for repeat testing had all tested negative by the Trombley assay (S2 Table).

In all, 211 WB specimens generated valid Xpert and Trombley results. Of these, 22/22 specimens that tested positive by the Trombley assay were positive by Xpert (sensitivity 100% [95% CI 84.6%–100%]) (Table 1), and 181/189 specimens that tested negative by the Trombley assay were negative by Xpert (specificity 95.8% [95% CI 91.8%–98.2%]), with the remaining 8/189 Trombley-negative specimens generating positive Xpert results (Table 1).

**Table 1:** Sensitivity and specificity of the Xpert Ebola assay versus the Trombley assay performed on clinical whole blood and buccal swab samples.

Sample Type	Sensitivity (Percent; 95% CI)	Specificity (Percent; 95% CI)	Adjusted Specificity* (Percent; 95% CI)
WB (*n* = 211)	22/22 (100%; 84.6%–100%)	181/189 (95.8%; 91.8%–98.2%)	181/182 (99.5%; 97.0%–100%)
BS (*n* = 64)	20/20 (100%; 83.2%–100%)	44/44 (100%; 92.0%–100%)	N/A

*Adjusted specificity is based on the resolution of seven discordant Trombley-negative/Xpert-positive tests in favor of Xpert based on prior Trombley-positive test results for those individuals.

N/A, not applicable.

**Fig 1:**
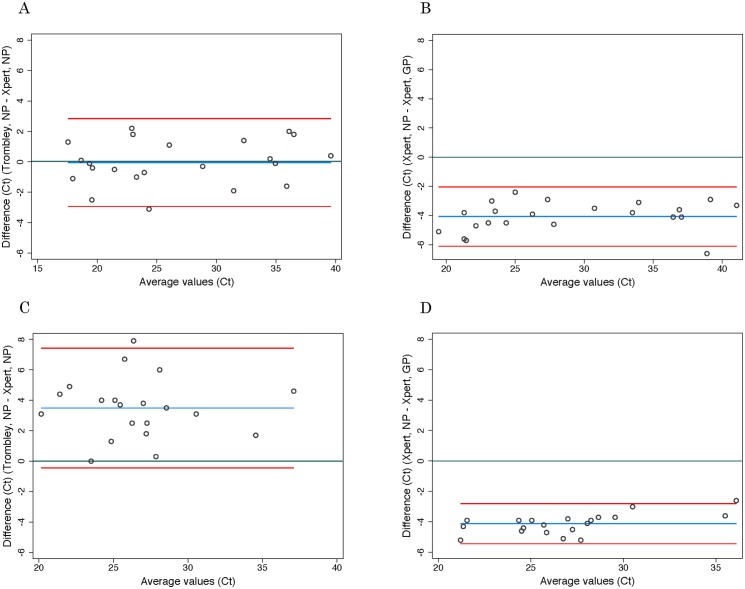
Evaluation of cycle threshold value agreement. Bland—Altman plots to evaluate agreement between cycle threshold values generated for the NP target by the Trombley versus Xpert assay for WB (A) and BS (C) samples and those generated by Xpert for the NP versus GP target for WB (B) and BS (D) samples. The blue line in each plot represents the “bias,” or average difference between the two methods. The red lines represent the 95% limits of agreement. The green line represents the line of no bias.

Ct values for WB specimens that tested positive by Trombley assay (NP gene target) ranged from 17.4 to 39.8 (mean 26.7, SD 7.2; median 24.0, IQR 19.4–34.7) (Table 2). Ct values for Xpert-positive WB specimens ranged from 16.9 to 43.4 (mean 29.8, SD 8.5; median 30.3, IQR 21.7–37.5) for the NP gene target and from 22.0 to 42.7 (mean 31.8, SD 7.2; median 29.5, IQR 24.9–39.0) for the GP gene target (Table 2). When comparing the results of both tests for concordant specimens, Ct values obtained for the NP gene target by Trombley and Xpert testing did not vary systematically (mean difference −0.04, 95% limits of agreement −2.93, 2.84; Fig 1A). For Xpert tests, Ct values obtained for the NP gene target were systematically lower than corresponding GP target Ct values (mean difference −4.06, 95% limits of agreement −6.09, −2.03; Fig 1B). For specimens yielding valid Xpert results, SPC Ct values ranged from 31.4 to 37.4 (mean 32.9, SD 2.5; median 32.7, IQR 32.4–33.3) and SAC Ct values ranged from 23.7 to 35.1 (mean 30.0, SD 1.6; median 30.3, IQR 29.4–30.9).

**Table 2:** Comparison of cycle threshold values for whole blood and buccal swab specimens that tested positive by Trombley and/or Xpert assay.

Specimen Type	Trombley/Xpert Test Agreement	Trombley NP Target	Xpert	
			NP Target	GP Target
**WB**	Concordant	21.2	21.7	25.4
	Concordant	37.1	35.1	38.7
	Concordant	26.6	25.5	30.1
	Concordant	22.8	23.8	26.2
	Concordant	33.0	31.6	35.4
	Concordant	17.4	18.5	24.1
	Concordant	22.8	25.9	28.8
	Concordant	37.4	35.6	42.2
	Concordant	19.4	19.8	24.5
	Concordant	34.6	34.4	38.5
	Concordant	18.3	20.8	25.3
	Concordant	23.6	24.3	28.2
	Concordant	18.7	18.6	24.3
	Concordant	24.0	21.8	24.8
	Concordant	19.3	19.4	23.2
	Concordant	18.2	16.9	22.0
	Concordant	30.5	32.4	35.5
	Concordant	39.8	39.4	42.7
	Concordant	35.1	36.7	N/A
	Concordant	23.9	22.1	26.6
	Concordant	28.7	29.0	32.5
	Concordant	34.9	35.0	39.1
	Discordant^a^	N/A	43.1	N/A
	Discordant^a^	N/A	41.8	N/A
	Discordant^a^	N/A	N/A	41.7
	Discordant^b^	N/A	N/A	42.1
	Discordant^a^	N/A	41.4	N/A
	Discordant^a^	N/A	37.7	40.6
	Discordant^a^	N/A	43.4	N/A
	Discordant^a^	N/A	38.6	N/A
**BS**	Concordant	23.5	23.5	28.2
	Concordant	25.5	24.2	29.3
	Concordant	31.1	25.1	28.9
	Concordant	28.0	27.7	31.4
	Concordant	39.4	34.8	37.4
	Concordant	28.9	25.1	30.3
	Concordant	21.7	18.6	23.8
	Concordant	35.4	33.7	37.3
	Concordant	28.5	26.0	30.1
	Concordant	27.3	23.6	27.8
	Concordant	32.1	29.0	32.0
	Concordant	27.5	25.0	29.5
	Concordant	26.2	22.2	26.8
	Concordant	29.1	22.4	26.8
	Concordant	24.5	19.6	23.5
	Concordant	28.1	26.3	30.2
	Concordant	30.3	22.4	26.3
	Concordant	30.3	26.8	30.5
	Concordant	27.1	23.1	27.0
	Concordant	23.6	19.2	23.5

Discordant test outcomes were Trombley-negative/Xpert-positive test results.

^a^Samples found to be from known EVD patients (i.e., patients who had tested positive by the Trombley assay on prior testing) who were receiving follow-up testing to monitor for clearance of viremia.

^b^The disease status of this patient is unknown.

N/A, not applicable (negative test result).

Further examination of the eight discordant specimens (Trombley-negative/Xpert-positive; Table 2) revealed that seven of these specimens were collected from known EVD patients (i.e., patients who had tested positive by the Trombley assay on prior testing) who were receiving follow-up testing to monitor for clearance of viremia. The Ct values obtained by Xpert testing for these seven discordant specimens were high (37.7–43.4 for the NP target, *n* = 6; 40.6–41.7 for the GP target, *n* = 2; Table 2), suggesting low-level viremia in the context of viral clearance. After resolving these seven discordant results in favor of Xpert, 181/182 true negatives were Xpert-negative (revised specificity of 99.5%, 95% CI 97.0%–100%; Table 1).

For Xpert testing, all 218 WB specimens were sampled by using a precision pipettor to add a 100 μl aliquot of EDTA-WB directly into the sample reagent; 84 of the specimens were also sampled by wicking EDTA-WB onto a swab and placing the swab into the sample reagent (see Methods). Five specimens generated invalid Xpert results in either the pipette- or swab-sampled test. Overall, 78/79 (98.7%) specimens with valid, paired pipette- and swab-sampled tests had concordant positive or negative Xpert results. The single discordant pair of results was from a Trombley-negative WB specimen that tested negative by Xpert for the pipette-sampled test and positive (NP Ct = 39.7, GP Ct = 41.8) for the swab-sampled test. Further examination of this specimen revealed that it was collected from a known EVD patient (i.e., previously Trombley-positive) who was being monitored for viral clearance. Ct values for positive specimens were well matched between the two sampling methods for both the NP (mean difference 0.45, SD 0.82) and GP (mean difference 0.10, SD 0.57) gene targets.

### Testing of Residual Clinical Buccal Swab Specimens

BS samples (*n* = 71) used for the study were submitted as part of the national postmortem surveillance program. Patient gender was recorded for 64 specimens, of which 28 (43.8%) were from female and 36 (56.3%) were from male patients. Patient age was recorded for 64 specimens, and ranged from 1 d to 96 y (mean 25.9 y, SD 28.6; median 10, IQR 1–50). Of the 71 BS specimens, 28 were fresh at the time of testing, and 43 were frozen and thawed. Of the fresh specimens, 17 (60.7%) were tested by Xpert and Trombley assays on the same day. The remaining fresh specimens were tested by the Xpert assay 1–2 d following Trombley testing (mean 1.64 d, SD 0.48; median 1, IQR 1–2). Frozen BS specimens were tested by Xpert and Trombley assays in parallel. Ambient temperatures in the FL during testing procedures ranged from 23.3 to 26.1°C (mean 26.3), and ambient humidity ranged from 60% to 69% (mean 64.9%).

Eight of the frozen BS specimens initially generated invalid Xpert results, all of which were due to SAC failure. SPC Ct values for these eight samples ranged from 32.0 to 33.5 (mean 32.5) (S2 Table), which was comparable to the SPC Ct values for BS specimens that generated valid Xpert results (range 31.2–36.0, mean 32.9). Adequate volume was available for repeat testing of six of these specimens, one of which generated a valid result that was included in subsequent analyses, and five of which again generated invalid results due to SAC failure (S2 Table).

Overall, 64 BS specimens (28 fresh and 36 frozen) generated valid Xpert and Trombley results. All of the 20 specimens that tested positive by Trombley assay were positive by Xpert (sensitivity 100%, 95% CI 83.2%–100%; Table 1), and all of the 44 specimens that tested negative by Trombley assay were negative by Xpert (specificity 100%, 95% CI 92.0%–100%; Table 1).

Ct values for BS specimens that tested positive by Trombley assay (18/20 of which were frozen specimens) ranged from 21.7 to 39.4 (mean 28.4, SD 4.0; median 28.1, IQR 25.7–30.3) (Table 2). Ct values for Xpert-positive BS specimens ranged from 18.6 to 34.8 (mean 24.9, SD 4.1; median 24.6, IQR 22.4–26.7) for the NP gene target and from 23.5 to 37.4 (mean 29.0, SD 3.7; median 29.1, IQR 26.8–30.5) for the GP gene target (Table 2). In contrast to WB specimens, Ct values for the NP gene target in BS specimens were consistently higher by Trombley assay than by Xpert testing (mean difference 3.49, 95% limits of agreement −0.44, 7.41; Fig 1C). Ct values obtained by Xpert testing for the NP gene target were again systematically lower than the corresponding Xpert GP Ct values (mean difference −4.11, 95% limits of agreement −5.43, −2.79; Fig 1D).

For all samples with valid test results, SPC Ct values were similar between fresh and frozen BS specimens, ranging from 31.2 to 35.0 (mean 32.6, SD 0.9; median 32.6, IQR 32.1–32.9) for fresh specimens and from 31.6 to 36.0 (mean 33.1, SD 0.9; median 32.9, IQR 32.6–33.5) for frozen specimens. In contrast, SAC Ct values tended to be higher in frozen than in fresh specimens, ranging from 24.8 to 33.9 (mean 28.9, SD 2.4; median 28.9, IQR 26.9–30.8) for fresh specimens and from 27.5 to 37.1 (mean 32.4, SD 2.8; median 32.7, IQR 29.7–35.0) for frozen specimens.

## Discussion

In this field study, the Cepheid GeneXpert assay performed on WB demonstrated 100% sensitivity and 95.8%–99.5% specificity compared to the benchmark Trombley assay performed on paired plasma samples. When the Xpert and Trombley assays were performed on BS samples, either fresh or frozen, the Xpert assay had 100% sensitivity and 100% specificity compared to the Trombley assay. The Trombley assay was used as the benchmark for this study because it was the clinical testing method employed by the PHE field biocontainment laboratory during the study period, and the assay has demonstrated excellent performance over the course of its deployment [4]. In RT-PCR-positive blood samples, Trombley (NP target) Ct values closely matched Xpert NP Ct values, despite the differences in samples tested (plasma versus WB). The WB sampling method used (conventional pipettor versus swab, both recommended in the Xpert Ebola package insert) did not appear to impact the results, and Ct values for samples collected using the two methods were roughly equivalent, despite the lower precision expected when sampling blood with a swab. Study operators found the swab sampling method to be straightforward and perhaps preferable to use of a pipettor given that pipette tips were treated as sharps in the laboratory and elimination of use of the pipettor reduced overall contamination risk.

Eight WB samples tested positive by the Xpert assay while their paired plasma samples were negative by the Trombley assay, leading to an initial specificity estimate of 95.8%. Notably, 7/8 of these samples were confirmed to be from patients who earlier in their disease had tested positive by the Trombley assay and were thus being monitored for clearance of viremia. This allowed the discordance to be resolved in favor of Xpert and raised the Xpert specificity estimate to 99.5%. The Xpert Ct values for these eight discordant samples ranged from 37.7 to 43.4, all at the upper range of detection for the assay. The Trombley internal control performed well in all eight discordant samples, indicating that inhibition of amplification was not a problem (of note, the Trombley internal control is an exogenous target [see Methods] that is spiked into the sample prior to nucleic acid extraction and thus does not depend on sample integrity). One potential explanation for the observed discordance is that the Xpert assay is more analytically sensitive than the Trombley assay; however, a direct comparison of the limit of detection of the two assays is not available. Interestingly, a recent publication comparing the performance of the BioFire FilmArray Ebola assays [6] to the US Centers for Disease Control and Prevention EBOV quantitative RT-PCR (qRT-PCR) assay suggests an alternative explanation. In that study, the authors identified clinical samples for which WB tested positive by both FilmArray and qRT-PCR, but the corresponding plasma prepared from the same sample tested negative on both assays. The authors interpreted these findings as “a trend toward the virus clearing from plasma before clearing from whole blood,” and suggested that this was potentially related to early infection of monocytes and was supported by precedents observed with infections with other RNA viruses. Future research should evaluate the hypothesis that plasma (as used in the Trombley assay) is potentially a less optimal sample type than WB for EVD testing, particularly in late stages of disease.

The Xpert platform offers advantages over conventional RT-PCR methods in its automated, integrated sample-to-answer configuration; this ease of use has allowed broad deployment in a variety of testing settings. However, the platform requirements are not trivial for deployment in truly resource-limited settings, given requirements for a constant supply of electricity and potentially for refrigeration of cartridges and other assay reagents. Moreover, despite the clear value of peripheral deployment of such a platform (i.e., outside of a field biocontainment laboratory setting), an operator with some laboratory expertise will still be required for platform and assay validation, quality control, and maintenance. Our experience indicated that troubleshooting by an experienced technician was required throughout the course of the study, including for evaluation of invalid results, stuck modules, and unexpected equipment shutdowns requiring platform reboot. It is difficult to imagine inexperienced operators coping with such issues without significant technical support. During our study, the failure of tests or entire runs for reasons unrelated to sample quality required repeat testing that in real practice would clearly impact result turnaround times. Finally, if implementation outside of a controlled laboratory setting is desired, the biosafety risks associated with loading cartridges outside containment must be carefully assessed. The Xpert Ebola assay is designed such that 20 min after a sample has been added to sample reagent, it is to be considered inactivated, and thus could be loaded into the cartridge outside of an FFI (“glove box”). The Xpert Ebola assay package insert (CE-IVD Xpert Ebola assay package insert, Revision A, June 2015) states that the sample reagent (2.5 ml) has been shown to completely inactivate 4.6 × 10^6^ PFU of added live EBOV, and in a published study [2] the reagent achieved at least a 6-log reduction of infectious EBOV. However, at the peak of EVD, viral load in blood can exceed 10^8^ RNA copies/ml [7]; given the challenge inherent in comparing viral RNA copies/ml and PFU/ml [2], it is unclear whether a 6-log “knock-down” may or may not still leave a small amount of viable virus in samples with very high viral load. Accordingly, in our study we chose to do all sample manipulation (including loading of cartridges with lysed and denatured samples) inside an isolator. Our laboratory staff also would have preferred to wipe cartridges down with 5,000 ppm sodium hypochlorite prior to removing them from the glove box and loading them on the Xpert platform, but the effects this might have on cartridge function are not yet known. All of these considerations have implications for operating the Xpert Ebola assay outside of a containment laboratory setting.

Our study had some limitations. First, sample processing methods were dictated by the use of excess clinical specimens for the study. Our BS processing procedure deviated from that intended in the package insert, in that we added aliquots of crude BS sample into the Xpert inactivation buffer, rather than adding the swab itself directly to the inactivation buffer immediately after collection. Additionally, we had to include a subset of frozen BS samples in our study in order to have sufficient numbers of positive samples for analysis, because of dropping EVD case rates over the course of the study. These samples contributed all of the invalid BS results. However, we anticipate that use of the intended Xpert processing protocol and fresh (rather than frozen) samples would further improve results over the excellent performance we observed here. Specifically, we would expect that placing the entire BS (with all of its cellular material, rather than just a fraction of it) directly into the inactivation buffer would increase assay yield, and that use of exclusively fresh BS specimens would lower the overall rate of invalid results. The thawing protocol used in this study may have contributed to the number of invalid BS tests, as 7/8 invalid results were observed in a batch of BS specimens that were thawed for 24 h at 4°C (possibly leading to increased degradation). However, suboptimal sample collection and handling prior to submission to the laboratory could also contribute to SAC failure; thus, invalid test rates will need to be evaluated in a larger study of fresh BS specimens submitted for routine testing.

The second limitation of this study was that we were ultimately unable to complete a planned point-of-care (POC) sample collection sub-study due to unanticipated changes in available clinical sites for our study. While sampling of WB with a swab in many ways simulates capillary blood collection and while our results suggest that the swab method works well in this assay, we cannot necessarily assume that capillary blood collected with a swab from a fingerstick will perform equivalently to venous WB sampled with a swab. However, three field studies thus far suggest that capillary blood does work well (in comparison to WB) for EVD diagnosis [4,8,9]. Overall, given that residual clinical WB and BS samples submitted to a working EVD field diagnostic laboratory were used for this study, the study population fully represents the population of interest.

There is general consensus that a highly sensitive and specific, less invasive (e.g., fingerstick or BS), cost-effective, POC diagnostic for EVD would be the ideal tool [10–12], but despite the explosion of test development activity in parallel with the outbreak response, it has been difficult to develop one test capable of meeting all of these goals. At the time of writing, ten assays have received US Food and Drug Administration emergency use authorization [13] and six have received WHO emergency use assessment and listing [14]; published data for field performance exist for only a few of these assays. The Corgenix lateral flow immunoassay for detection of EBOV VP40 matrix protein performed very well for fingerstick testing in a recent field study [4] and satisfies additional critical goals of low cost and POC use, but its sensitivity and specificity were lower than that of benchmark RT-PCR assays performed in parallel (including the Trombley assay used in this study), leading to controversy around optimal deployment [15]. The BioFire FilmArray sample-to-answer molecular assays (BioFire FilmArray BioThreat panel and FilmArray BT E-panel) have the advantage of requiring no refrigeration and performed well in three recent studies [6,16,17] compared to the EBOV RT-PCR assays developed by the US Centers for Disease Control and Prevention [6,16] or the Trombley assay used by PHE [17]. However, the analytical sensitivity compared to the Xpert Ebola test was inferior [2]. The FilmArray platform is not designed for high-throughput testing, potentially limiting its deployment. The Cepheid Xpert Ebola assay has some advantages over these other diagnostics: (a) higher throughput (multiple modules per platform) while maintaining a low-complexity sample-to-answer format; (b) highly accurate performance, as evidenced in this study; and (c) relatively rapid turnaround time (we estimate from our experience that time from sample receipt to result would be ~2.5 h, versus ~4 h for the Trombley assay). However, it remains to be seen whether the test can truly be deployed at POC (or near POC) and in what types of facilities; moreover, whether the performance of the assay in fingerstick samples is equivalent to its performance in WB should be formally evaluated, as should its performance with a larger set of fresh BS samples. As noted above, additional questions remain regarding complete inactivation of virus in samples with very high viral loads (and thus about optimal biosafety practices for loading cartridges); requirements for refrigeration, uninterrupted electricity, and platform validation and maintenance must be considered; and, finally, test cost must be considered and potentially negotiated to allow wide deployment in areas of maximal need. In sum, our data indicate that the Xpert Ebola assay has excellent performance in a field laboratory setting using both WB and BS specimens and thus provides the opportunity for highly accurate, rapid sample-to-answer diagnosis of EVD; future operational research should examine the spectrum of clinical testing scenarios to determine which are feasible for Xpert Ebola assay deployment.

## Supporting Information

S1 Dataset Study dataset. (XLSX)

S1 Table STARD checklist. (DOCX)

S2 Table Internal control data for whole blood and buccal swab samples with Xpert results of “invalid,” “error,” or “no result.” (DOCX)

S1 Text Study protocol. (DOCX)

## Abbreviations

BS: buccal swab
CI: confidence interval
Ct: cycle threshold
EBOV: Ebola virus
EVD: Ebola virus disease
FFI: flexible film isolator
FL: field laboratory
GP: glycoprotein
IQR: interquartile range
NP: nucleoprotein
POC: point of care
qRT-PCR: quantitative reverse transcription PCR
RT-PCR: reverse transcription PCR
SAC: sample adequacy control
PHE: Public Health England
SPC: sample processing control
SD: standard deviation
WB: whole blood

## References

[1] Boehme CC, Nabeta P, Hillemann D, Nicol MP, Shenai S, Krapp F, et al Rapid molecular detection of tuberculosis and rifampin resistance. N Engl J Med. 2010;363:1005–1015. 10.1056/NEJMoa0907847 20825313 PMC2947799

[2] Pinsky BA, Sahoo MK, Sandlund J, Kleman M, Kulkarni M, Grufman P, et al Analytical performance characteristics of the Cepheid GeneXpert Ebola assay for the detection of Ebola virus. PLoS ONE. 2015;10:e0142216 10.1371/journal.pone.0142216 26562786 PMC4643052

[3] Trombley AR, Wachter L, Garrison J, Buckley-Beason VA, Jahrling J, Hensley LE, et al Comprehensive panel of real-time TaqMan polymerase chain reaction assays for detection and absolute quantification of filoviruses, arenaviruses, and New World hantaviruses. Am J Trop Med Hyg. 2010;82:954–960. 10.4269/ajtmh.2010.09-0636 20439981 PMC2861391

[4] Broadhurst MJ, Kelly JD, Miller A, Semper A, Bailey D, Groppelli E, et al ReEBOV Antigen Rapid Test kit for point-of-care and laboratory-based testing for Ebola virus disease: a field validation study. Lancet. 2015;386:867–874. 10.1016/S0140-6736(15)61042-X 26119838

[5] Bland JM, Altman DG. Measuring agreement in method comparison studies. Stat Methods Med Res. 1999;8:135–160. 10501650 10.1177/096228029900800204

[6] Southern TR, Racsa LD, Albarino CG, Fey PD, Hinrichs SH, Murphy CN, et al Comparison of FilmArray(R) and qRT-PCR for the detection of Zaire Ebolavirus from contrived and clinical specimens. J Clin Microbiol. 2015;53:2956–2960. 26157148 10.1128/JCM.01317-15 PMC4540924

[7] Schieffelin JS, Shaffer JG, Goba A, Gbakie M, Gire SK, Colubri A, et al Clinical illness and outcomes in patients with Ebola in Sierra Leone. N Engl J Med. 2014;371:2092–2100. 10.1056/NEJMoa1411680 25353969 PMC4318555

[8] Strecker T, Palyi B, Ellerbrok H, Jonckheere S, de Clerck H, Bore JA, et al Field evaluation of capillary blood samples as a collection specimen for the rapid diagnosis of Ebola virus infection during an outbreak emergency. Clin Infect Dis. 2015;61:669–675. 10.1093/cid/civ397 25991465 PMC4530726

[9] Walker NF, Brown CS, Youkee D, Baker P, Williams N, Kalawa A, et al Evaluation of a point-of-care blood test for identification of Ebola virus disease at Ebola holding units, Western Area, Sierra Leone, January to February 2015. Euro Surveill. 2015;20(12). 25846490 10.2807/1560-7917.es2015.20.12.21073

[10] Chua AC, Cunningham J, Moussy F, Perkins MD, Formenty P. The case for improved diagnostic tools to control Ebola virus disease in West Africa and how to get there. PLoS Negl Trop Dis. 2015;9:e0003734 10.1371/journal.pntd.0003734 26068890 PMC4465932

[11] Butler D. Ebola experts seek to expand testing. Nature. 2014;516:154–155. 10.1038/516154a 25503213

[12] World Health Organization. Target product profile for Zaire ebolavirus rapid, simple test to be used in the control of the Ebola outbreak in West Africa. 2014 Oct 3 [cited 10 Mar 2015]. Available: http://www.finddiagnostics.org/programs/ebola/product_development/tpp.html.

[13] US Food and Drug Administration. Emergency use authorization. [cited 23 Nov 2015]. Available: http://www.fda.gov/EmergencyPreparedness/Counterterrorism/MedicalCountermeasures/MCMLegalRegulatoryandPolicyFramework/ucm182568.htm.

[14] World Health Organization. Emergency use assessment and listing (EUAL) procedure for Ebola virus disease (IVDs). [cited 23 Nov 2015]. Available: http://www.who.int/diagnostics_laboratory/procurement/purchasing/en/.

[15] Broadhurst MJ, Semper A, Bailey D, Pollock NR. ReEBOV Antigen Rapid Test kit for Ebola—authors’ reply. Lancet. 2015;386:2255–2256. 26681290 10.1016/S0140-6736(15)01109-5

[16] Leski TA, Ansumana R, Taitt CR, Lamin JM, Bangura U, Lahai J, et al Use of the FilmArray System for detection of Zaire ebolavirus in a small hospital in Bo, Sierra Leone. J Clin Microbiol. 2015;53:2368–2370. 10.1128/JCM.00527-15 25972415 PMC4473222

[17] Weller SA, Bailey D, Matthews S, Lumley S, Sweed A, Ready D, et al Evaluation of the Biofire FilmArray BioThreat-E Test (v2.5) for rapid identification of ebola virus disease in heat-treated blood samples obtained in Sierra Leone and the United Kingdom. J. Clin Microbiol. 2016;54:114–119. 10.1128/JCM.02287-15 26537445 PMC4702715

