# Supplementary material for: Performance of the GeneXpert Ebola Assay for Diagnosis of Ebola Virus Disease in Sierra Leone: A Field Evaluation Study
Source: PLoS Med. 2016 Mar 29;13(3):e1001980. doi: 10.1371/journal.pmed.1001980 (PMC4811569; doi:10.1371/journal.pmed.1001980)
Supplement: S2 Table — (DOCX) [file pmed.1001980.s003.docx]

**S2 Table**: Internal control data for whole blood and buccal swab samples with Xpert results of ‘invalid’, ‘error’ or ‘no result’.

| **Study #** | **Trombley** | | **Xpert Result** | **SAC** | | **SPC** | | **Error description** |
| --- | --- | --- | --- | --- | --- | --- | --- | --- |
|  | **Result** | **Ct** |  | **Result** | **Ct** | **Result** | **Ct** |  |
| **Whole blood** | | | | | | | | |
| 007 | NEG | N/A | INVALID | PASS | 32.8 | FAIL | 0.0 | - |
| 008 | POS | 37.1 | NO RESULT | N/A | 0.0 | N/A | 0.0 | Error 2025: System failed to find the plunger home position |
| 008 RPT* | POS | 37.1 | POS | NA | 29.3 | NA | 32.7 | - |
| 031 | NEG | N/A | INVALID | FAIL | 0.0 | FAIL | 0.0 | - |
| 046 | NEG | N/A | INVALID | PASS | 31.1 | FAIL | 0.0 | - |
| 096 | NEG | N/A | INVALID | FAIL | 0.0 | PASS | 32.3 | - |
| 097 | NEG | N/A | ERROR | NO RESULT | 0.0 | NO RESULT | 0.0 | Error 5007: Probe check failed [SPC, SAC, GP] |
| 160 | NEG | N/A | ERROR | NO RESULT | 0.0 | NO RESULT | 0.0 | Error 5007: Probe check failed [GP] |
| 161 | NEG | N/A | NO RESULT | NO RESULT | 0.0 | NO RESULT | 0.0 | Error 2016: Unable to find valve home position |
| 161 RPT** | NEG | N/A | NEG | PASS | 29.3 | PASS | 32.6 | - |
| 187 | NEG | N/A | ERROR | NO RESULT | 0.0 | NO RESULT | 0.0 | Error 2005: motion of syringe drive not detected |
| **Buccal swab^$^** | | | | | | | | |
| 343 | NEG | N/A | INVALID | FAIL | 0.0 | PASS | 32.2 | - |
| 358 | NEG | N/A | INVALID | FAIL | 0.0 | PASS | 32.6 | - |
| 358 RPT | NEG | N/A | INVALID | FAIL | 0.0 | PASS | 32.6 | - |
| 359 | NEG | N/A | INVALID | FAIL | 0.0 | PASS | 32.3 | - |
| 359 RPT | NEG | N/A | INVALID | FAIL | 0.0 | PASS | 32.6 | - |
| 362 | NEG | N/A | INVALID | FAIL | 40.4 | PASS | 32.8 | - |
| 362 RPT | NEG | N/A | INVALID | FAIL | 38.8 | PASS | 32.3 | - |
| 364 | NEG | N/A | INVALID | FAIL | 36.7 | PASS | 32.3 | - |
| 365 | NEG | N/A | INVALID | FAIL | 36.5 | PASS | 33.5 | - |
| 365 RPT** | NEG | N/A | NEG | PASS | 35.8 | PASS | 32.6 | - |
| 367 | NEG | N/A | INVALID | FAIL | 37.4 | PASS | 32.0 | - |
| 367 RPT | NEG | N/A | INVALID | FAIL | 37.3 | PASS | 32.2 | - |
| 368 | NEG | N/A | INVALID | FAIL | 40.8 | PASS | 32.7 | - |
| 368 RPT | NEG | N/A | INVALID | FAIL | 0.0 | PASS | 32.8 | - |

SAC, Sample Adequacy Control. SPC, Sample Processing Control. Ct, cycle threshold value. RPT, repeat (i.e., repeat testing on the same sample).

*On repeat testing the Xpert result for this sample was ‘Ebola GP detected’ (Ct 38.7) and ‘Ebola NP detected’ (Ct 35.1).

**On repeat testing the Xpert result for these samples was ‘Ebola GP not detected’ (Ct 0.0) and ‘Ebola NP not detected’ (Ct 0.0).

^$^Sample #343 was thawed for 30 min at room temperature; all other failed buccal swab samples were thawed for 24 hrs at 4^o^C.
