# Supplementary material for: Performance of the GeneXpert Ebola Assay for Diagnosis of Ebola Virus Disease in Sierra Leone: A Field Evaluation Study
Source: PLoS Med. 2016 Mar 29;13(3):e1001980. doi: 10.1371/journal.pmed.1001980 (PMC4811569; doi:10.1371/journal.pmed.1001980)
Supplement: S1 Text — (DOCX) [file pmed.1001980.s004.docx]

**Study Title:** **Evaluation of the performance of novel rapid diagnostics for Ebola Virus Disease at point-of-care in Sierra Leone**

**Principal Investigators:** Megan Murray, M.D., Ph.D., Brigham and Women’s Hospital, Boston, MA; Nira Pollock, M.D., Ph.D., Boston Children’s Hospital, Boston MA

**Co-Investigators:** M. Jana Broadhurst, M.D., Ph.D., Partners in Health; J. Daniel Kelly, M.D., Partners In Health and Wellbody Alliance, Sierra Leone; Ann C. Miller, Ph.D., M.P.H., Harvard Medical School, Boston, MA

**CLINICAL STUDY SUMMARY**

**Primary Objective:** The purpose of this study is to evaluate the performance of newly-developed Ebola Zaire Virus (EBOV) rapid diagnostic tests for point-of-care (POC) diagnosis of Ebola virus disease (EVD). Specifically, we will compare the performance of novel POC tests [for detection of EBOV nucleic acid or antigen, as performed at point-of-care (POC)] to gold standard testing [RT-PCR, as performed in a reference laboratory] in suspected EVD cases presenting to Partners in Health (PIH) clinical sites in Sierra Leone. We will evaluate each novel POC test in a separate, dedicated evaluation (substudy) to facilitate rapid evaluation of test performance; each substudy will have the same design, as outlined below. *Results of novel POC tests will not be used for clinical management.*

**GENERAL STUDY INFORMATION**

This study is designed to comply with the Pharmacy Board of Sierra Leone Good Clinical Practice guideline and the Pharmacy Board of Sierra Leone Guideline for Conducting Clinical Trial of Medicines, Vaccines and Medical Devices.

In addition, it is designed to comply with applicable sections of current Good Clinical Practices (cGCPs) following the regulations, standards, and guidance documents:

- 21 CFR Part 50, Protection of Human Subjects
- 21 CFR Part 54, Financial Disclosure by Clinical Investigators
- 21 CFR Part 56, Institutional Review Board
- 21 CFR Part 812, Investigational Device Exemptions
- Guidance for Industry: Investigator Responsibilities, Protecting the Rights, Safety, and Welfare of Study Subjects

Study Sponsor(s): Support for PIH Ebola research activities is provided by a prior gift from the Stephen Kahn Foundation. The manufacturer of each novel POC EBOV test will gift sufficient quantities of the test/platform for training and test evaluation activities required for that specific sub-study. The first novel POC tests to be studied under this protocol are manufactured by Cepheid (Sunnyvale, CA) and Corgenix (Broomfield, CO) [see Appendix (Section 12)]. Any provision of additional funds needed to support study activities will be negotiated with the test manufacturer on a case-by-case basis. The Foundation for Innovative New Diagnostics (FIND) may also provide research support for individual sub-studies on a case-by-base basis.

Sponsor Liaison: Nira Pollock, MD, PhD, Boston Children’s Hospital (study co-PI)

IRB: Partners Human Research Committee, Boston, MA; Sierra Leone Scientific Review and Ethics Committee

Performance Site: Partners in Health Ebola Treatment Units and Community Care Centers, Sierra Leone; designated affiliated reference laboratories serving PIH units

1. **BACKGROUND AND SIGNIFICANCE**
   1. Historical background

The aim of this study is to validate novel rapid POC tests that can determine if a person has EVD, a serious and often fatal hemorrhagic disease. Persons with EVD may develop severe vomiting, diarrhea, severe electrolyte abnormalities, shock, multi-organ dysfunction, and sometimes bleeding from the mouth, nose, eyes, or rectum.

The largest and most widespread outbreak of EVD continues to spread through West Africa, with over 20,000 cases reported in Guinea, Sierra Leone, Liberia, Senegal and Nigeria as of January 7, 2015.[^1^](#_ENREF_1) The possibility of global spread of the disease was realized recently with the diagnosis of EVD in patients in the United States and Spain.[^2^](#_ENREF_2) The EVD outbreak appears to have originated near the town of Guéckédou, which is in the forest region of Guinea and close to the borders of Sierra Leone and Liberia.[^3^](#_ENREF_3)^,^[^4^](#_ENREF_4) Sequence analyses indicated that the West African Ebola virus variant diverged from Middle African lineages approximately a decade ago.[^5^](#_ENREF_5) Genetic similarity across the 2014 samples suggested a single introduction from an animal reservoir with human-to-human transmission sustaining the outbreak.

EBOV infection presents many challenges to clinical management.[^6^](#_ENREF_6)^,^[^7^](#_ENREF_7) Case fatality rates (CFRs) in past EVD outbreaks have ranged from 45% to 90%.[^8^](#_ENREF_8) Exposure to EBOV-infected patients is a hazard for health care providers, one that has been exacerbated by the scale of the current outbreak. There are currently no approved treatments for EVD, although early and aggressive supportive care, including intravenous fluids and empiric antibiotics, is thought to improve survival. Care in advanced settings may increase survival by facilitating maintenance of hydration, circulatory volume, and blood pressure.[^6^](#_ENREF_6)^,^[^9^](#_ENREF_9)

In the absence of a vaccine or effective treatment, prevention remains the mainstay of epidemic control. This includes early isolation of infectious cases and safe and dignified burials.

EBOV testing is critical for early case identification and isolation. In the current epidemic, laboratory testing is based on high-complexity nucleic acid amplification methods performed in centralized laboratory units, creating a number of challenges to early diagnosis and isolation. First, the turnaround time between specimen collection and receiving results is slow. While the PCR testing itself takes many hours from start to finish, the larger barriers to efficient diagnosis in remote areas are the complex logistics of actually getting the sample to the laboratory (requiring dedicated transport, often over difficult terrain) and having results return efficiently. In much of Sierra Leone, the time between testing and receiving results is currently 4-5 days or more. Second, Ebola suspects are kept in ‘holding centers’ while awaiting these results, creating additional risks of cross-infection. In many such centers, beds are currently filled to capacity while more than 50% of patients test EBOV negative. Third, the need for specialized laboratories creates barriers to decentralized testing. Suspects from remote areas are less likely to present for testing and may remain at home for longer periods, further accelerating household transmission.

In past outbreaks, EVD has been characterized by a constellation of signs and symptoms beginning with fever and progressing to include diarrhea, vomiting and, in a subset of patients, hemorrhage.[^7^](#_ENREF_7)^,^[^9^](#_ENREF_9)^,^[^10^](#_ENREF_10) However, the sporadic nature of EBOV outbreaks and their occurrence in remote resource-limited settings has precluded acquisition of extensive clinical and laboratory data. The natural history of EVD in the current outbreak may vary from that in prior outbreaks.

As of January, 2015, the Partners in Health (PIH) clinical teams are running two Ebola Treatment Units (ETUs) and seven community care centers (CCCs) in the Port Loko surrounds, and four CCCs in the Kono surrounds, with more CCCs actively being constructed. For diagnosis of suspected EVD by standard RT-PCR, the PIH Port Loko units are currently sending venous whole blood to a reference laboratory in Port Loko run by Public Health England. The PHE RT-PCR assay is one of the assays favored by the WHO as a benchmark for method comparisons. Confirmatory RT-PCR testing in Kono is currently unavailable, but a reference laboratory furnished by the Dutch government will be operating in Koidu Town later in January with analytic protocols for EBOV detection that closely resemble those of PHE.

- 1. Previous pre-clinical or clinical studies leading up to, and supporting the proposed research

Novel POC tests evaluated under this protocol will have each undergone rigorous analytical testing with simulated and/or banked samples prior to selection for clinical validation under this protocol, and will have been vetted through extensive discussions with leadership of the WHO/FIND Ebola Diagnostics Access Collaboration (EDAC). A summary of performance data available for each test selected is described in section Vc.

- 1. Rationale behind the proposed research, and potential benefits to patients and/or society

EBOV testing using rapid POC tests holds great potential. A test that can reliably detect early infection at POC can facilitate rapid case identification and isolation; effectively triage confirmed cases to Ebola Care Centers and Treatment Units, where early care has been associated with improved survival; open up bed-space currently occupied by non-Ebola cases; reduce the risks of cross-infection; more readily identify EBOV deaths for safe burial; and make decentralized EBOV testing data more readily available to inform prevention measures.

Processes for venous blood draw for diagnostic testing at PIH sites have been closely scrutinized to ensure that they meet CDC and WHO biosafety standards. Technicians (local health care workers) drawing blood are trained specifically in venipuncture and in packaging venipuncture whole blood for transport to reference laboratories, and are wearing appropriate personal protective equipment (PPE) at the time of blood draw and sample handling. These technicians are in an optimal position to perform the novel tests under study at POC, including performing the fingerprick, collecting that fingerprick sample, and performing the novel test using that fingerprick sample. They are also in an optimal position to collect alternative sample types (saliva/buccal swab, urine) and perform the POC test on that sample type. All novel POC tests will be performed within the “red zones” in the Ebola care facilities, meaning that everyone in that area will similarly already be wearing appropriate PPE per clinical routine, and the testing will not add any new biosafety hazard to routine clinical practices. Moreover, the processes used for study testing will thus mirror processes that would be optimal for future implementation of POC clinical testing for Ebola in the same care facilities.

In short, the existing infrastructure for clinical care at PIH sites in Sierra Leone provides excellent opportunities to evaluate novel POC tests for diagnosis of EVD.

1. **SPECIFIC AIMS**
   1. Specify objectives and hypotheses to be tested in the research

**Primary Objective**: The purpose of this study is to evaluate the performance of newly-developed Ebola Zaire Virus (EBOV) rapid diagnostic tests for point-of-care (POC) diagnosis of Ebola virus disease (EVD). Specifically, we will compare the performance of novel POC tests [for detection of EBOV nucleic acid or antigen, as performed at point-of-care (POC)] to gold standard testing [RT-PCR, as performed in a reference laboratory] in suspected EVD cases presenting to Partners in Health (PIH) clinical sites in Sierra Leone. We will evaluate each novel POC test in a separate, dedicated evaluation (substudy) to facilitate rapid evaluation of test performance; each substudy will have the same design, as outlined below. *Results of novel POC tests will not be used for clinical management.* For each novel POC test evaluated, we will investigate the following aims:

***Aim 1:*** Validate sensitivity and specificity of the novel POC EBOV test, as performed at POC on capillary blood obtained via fingerprick, vs gold-standard real-time PCR (RT-PCR), as performed in an affiliated reference laboratory on venous blood. Venous whole blood will also be tested on the novel POC EBOV test in the reference laboratory as a control.

***Aim 2*:** Evaluate feasibility and ease-of-use of the novel POC EBOV test at POC.

**Exploratory Aim 1.** Validate sensitivity and specificity of the novel POC EBOV test, as performed at POC on a saliva/buccal swab sample, vs gold-standard RT-PCR, as performed in an affiliated reference laboratory on venous blood.

**Exploratory Aim 2.** Validate sensitivity and specificity of the novel POC EBOV test, as performed at POC on urine, vs gold-standard RT-PCR, as performed in an affiliated reference laboratory on venous blood.

**Hypothesis:** Each novel EBOV rapid diagnostic test, when performed at POC on a fingerprick sample, will maintain clinical performance of >90% sensitivity and >95% specificity when compared to RT-PCR performed at an affiliated, WHO-approved reference laboratory in Sierra Leone.

1. **SUBJECT SELECTION**
   1. Inclusion/exclusion criteria

**Inclusion Criteria:**

- Any person presenting to participating Partners in Health sites in Sierra Leone who meets the WHO definition of a suspected EVD case, in accordance with the following screening protocol designated by the Ministry of Health:

Any person suffering or having suffered from a sudden onset of fever and having had contact with suspected, probable or confirmed Ebola case,

OR:
Any person with sudden onset of fever and at least three of the following symptoms:

- Headaches
- Anorexia/loss of appetite
- Lethargy
- Aching muscles or joints
- Breathing difficulties
- Vomiting
- Diarrhea
- Stomach pain
- Difficulty swallowing
- Hiccups

OR

Any person with inexplicable bleeding

**Exclusion Criteria:**

**-** Hemodynamic instability as determined by the treating physician

- Patient unable to cooperate with fingerprick and/or venous blood draw

- Patient unable to give verbal consent

- 1. Source of subjects and recruitment methods

This study will enroll suspected EVD patients presenting to participating PIH sites in Sierra Leone. Recruitment will occur at the time that the patient is approached for venipuncture to obtain blood for EVD testing per clinical routine. Study staff wearing full PPE (accompanied by the technician performing phlebotomy at that PIH site, per clinical routine) will approach potential participants in the screening area of PIH-run treatment centers to inform them of the study. Interested patients will then be consented and enrolled immediately prior to venous blood draw for EBOV testing.

1. **SUBJECT ENROLLMENT**
   1. Methods of enrollment, including procedures for patient registration and/or randomization

We will enroll up to 2,000 suspected EVD patients at all sites (up to 400 consented subjects per substudy, up to 5 substudies; see also Section 3, “sample size analysis”) and will test an additional 1000 (maximum) discarded specimens in pilot studies preceding POC testing in each substudy. Samples collected specifically for POC testing (fingerprick, saliva/buccal swab, and/or urine) will be collected with informed consent. Both males and females of all ages are eligible for enrollment. The distribution of testing will be as follows:

| Testing site | Samples obtained | Enrollment/samples Total |
| --- | --- | --- |
| POC Testing sites  (consent obtained) | Capillary whole Blood, saliva/buccal swab, and/or urine (POC testing); Venous Whole Blood (testing in reference lab) | 2000 (consented subjects; up to 400 per sub-study) |
| De-identified Sample Testing  (pilot testing of novel rapid test in reference laboratory, to precede each POC substudy; no consent obtained) | Venous Whole Blood | 1000 (discarded/excess specimens; up to 200 per sub-study) |
| Total |  | 3,000 |

- 1. Procedures for obtaining informed consent (including timing of consent process)

At centralized testing centers (reference laboratories) using excess venous whole blood samples for this study, no consent will be obtained.

Eligible potential subjects will be recruited and witnessed, documented verbal informed consent will be obtained by study staff trained in human subjects protections at sites conducting sample collection for novel POC test performance. Recruitment will occur at the time that the patient is approached for venipuncture to obtain blood for EVD testing per clinical routine. Due to infection control restrictions and low levels of literacy, we cannot obtain written consent. Due to the severity and high mortality rate of EVD and concerns for transmission, strict infection control measures are in place at all EVD treatment and holding centers. These measures require that all items coming in contact with suspected EVD patients be incinerated, preventing the collection of signed consent forms. A verbal consent script describing the study purpose and procedures will be read to each subject. For patients aged <18 years, verbal informed consent will be obtained from a parent or guardian. Verbal assent will be obtained from patients aged 13-17 years. An informed consent/assent log will document study subjects who verbally consent to enroll in the study. This log will be stored in hard copy in a locked cabinet and/or a password-protected document on an encrypted computer in Sierra Leone.

- 1. Treatment assignment and randomization (if applicable)

Our intention is that no subject will participate in more than one substudy, and that a given site (e.g. CCC) will only be evaluating one test (i.e. executing one substudy) at a time. However, given the urgency of completing these critical test evaluations during the current outbreak, in the event of precipitously dropping case loads or unforeseen logistic constraints we will consider inviting a given subject to participate in more than one substudy in parallel. Under such circumstances the patient would be consented twice (once per substudy) and would accordingly receive two fingersticks.

1. **STUDY PROCEDURES**
   1. Study visits and parameters to be measured

Division of testing activities

1. POC testing sites

Samples will be collected by fingerprick, buccal swab, and/or standard urine collection techniques for POC testing, as appropriate to the novel POC test under evaluation. Venipuncture samples will be collected in parallel for confirmatory testing at a central reference laboratory (below), per clinical routine at the selected PIH site. Verbal consent will be obtained from all subjects whose samples will be used for POC testing.

1. Central Reference Laboratory

A. Pilot discarded/excess specimen study: Prior to initiation of POC testing, the designated central reference laboratory participating in the substudy will perform a brief and focused evaluation of the novel rapid test using a limited number of discarded/excess venipuncture whole blood specimens in order to confirm that performance of the novel rapid test meets expectations. Specifically, whole blood specimens sent to this laboratory for testing by RT-PCR per clinical routine will also be used to test the novel POC test in parallel. These specimens will come from multiple clinical sites. After RT-PCR testing is performed per clinical routine, excess whole blood specimen will be de-identified and used for the rapid POC test, allowing direct comparison of results of testing by RT-PCR and the novel POC test. Consent will not be obtained for use of these specimens.

B. Clinical substudy: In parallel with POC testing, confirmatory testing of venipuncture blood will be performed at a designated central reference laboratory by benchmark RT-PCR, per clinical routine at the selected PIH site. These RT-PCR results will be used for clinical management, as per existing clinical routine. After RT-PCR testing is performed, excess whole blood specimen will be de-identified and used for testing with the POC test in the reference laboratory.

Note: Our current designated reference laboratory for the Port Loko PIH sites is the PHE laboratory in Port Loko, and for the Kono PIH sites will be a reference laboratory furnished by the Dutch government that will be operative in Koidu Town in mid-late January 2015 (with analytic protocols for EBOV detection that closely resemble those of PHE). Details regarding reference lab utilization of discarded (deidentified, excess) clinical specimens are being discussed with the WHO/FIND leadership (EDAC) and reference lab leadership, and will also be discussed with the Laboratory Technical Working Group and Surveillance Pillars in Sierra Leone.

- 1. Drugs to be used

This project will not involve additions or changes to therapeutic regimens. Results of novel POC tests will not be used for clinical management.

- 1. Devices to be used

A. ReEBOV^TM^ Rapid Diagnostic Test

i. Description. The ReEBOV^TM^ Rapid Diagnostic Test (Corgenix, Broomfield, CO) is a recombinant Lateral Flow Immunoassay (LFI) for detection of the EBOV VP-40 protein in whole blood.

The ReEBOVTM Antigen Rapid Test is performed as a dipstick immunoassay. Whole blood (fingerprick or venous collection) is added to the sample pad and the dipstick is then added to a test tube containing sample buffer, which initiates flow of sample through the reagent pads and across the nitrocellulose membrane. As the assay develops, EBOV VP40 antigen forms immune-complexes with anti-EBOV VP40 polyclonal antibody gold conjugate. As these complexes are captured by the anti-EBOV VP40 polyclonal antibody Test Line, the deposition of the gold nanoparticles generates a pink to red signal which corresponds to the concentration of EBOV VP40 antigen in the sample. Excess gold-conjugate is captured by the Control Line, indicating a valid result. Visual interpretation is made after 5 to 15 minutes of development time.

ii. Analytical performance data. During preliminary evaluation against stored serum specimens in Kenema, Sierra Leone, the ReEBOV^TM^ Rapid Diagnostic Test demonstrated good agreement with the comparator method (CDC PCR assay as previously described[^11^](#_ENREF_11)). The rapid test gave 91% agreement with PCR-positive specimens and 93% agreement with PCR-negative specimens in 83 total specimens. The positive predictive value was 93%, while the negative predictive value was 91%. Signal range (band intensity) and uniformity were satisfactory.

The test was subsequently optimized to improve specificity; updated performance data is not currently available as the test is undergoing review for FDA Emergency Use Authorization (EUA) approval.

iii. Sample types to be tested at POC: capillary whole blood (fingerprick)

B. GeneXpert Ebola

i. Description. The Cepheid^®^ (Sunnyvale, CA) GeneXpert^®^ Ebola Assay, performed on the GeneXpert Instrument Systems, is a qualitative nucleic acid amplification test designed to rapidly detect Zaire Ebola virus total nucleic acids on the automated GeneXpert® Systems using human whole blood (venipuncture or fingerstick) and buccal cheek swabs (saliva). The GeneXpert Ebola Assay is a rapid, automated test performed on the Cepheid GeneXpert Dx System.

The GeneXpert Instrument Systems automate and integrate sample purification, nucleic acid amplification, and detection of the target sequence in simple or complex samples using real-time reverse transcription PCR (RT-PCR). The systems consist of an instrument, personal computer, and preloaded software for running tests and viewing the results. The systems require the use of single-use disposable GeneXpert cartridges that hold the RT-PCR reagents and host the RT-PCR processes. Because the cartridges are self-contained, cross-contamination between samples is minimized.

The GeneXpert Ebola Assay includes reagents for the detection of Zaire Ebola virus total nucleic acids in specimens as well as a sample adequacy control and an internal control to ensure adequate addition of sample and processing of the target and to monitor for the presence of inhibitor(s) in the RT and PCR reactions. The Probe Check Control (PCC) verifies reagent rehydration, PCR tube filling in the cartridge, probe integrity, and dye stability.

The GeneXpert platform is already in wide use around the world; in particular the Xpert MTB-RIF assay is being used in many settings with ambient temperature and humidity similar to that typical in SL.

ii. Analytical performance data. Xpert Ebola utilizes two targets for maximum sensitivity and to minimize the risk for false negatives due to sequence variants. Limit of detection is below 300 copies / ml as determined by using Ebola RNA as target. The assay utilizes one internal control as well as a human housekeeping gene to ensure that an adequate sample has been collected and processed appropriately inside the cartridge.

iii. Sample types to be tested at POC: capillary whole blood (fingerprick), buccal swab

**NOTE**: to date, we have selected two novel POC tests for the first two substudies [see above; this information is located in the Appendix of the protocol as submitted to the Sierra Leone Scientific Review and Ethics Committee]; as additional novel POC tests are selected for study, details for those tests will be added to the protocol and resubmitted to all review boards as a protocol amendment. Total sample size is based on a plan to evaluate up to 5 different novel POC tests in 5 separate substudies.

- 1. Procedures/surgical interventions, etc.

This project will not involve surgical procedures or interventions.

- 1. Data to be collected and when the data is to be collected

*Capillary Blood, Saliva/buccal swab, and/or Urine Testing, at PIH clinical sites*: We will enroll suspected EVD cases that present to specified sites and will obtain documented verbal informed consent prior to collecting fingerprick blood, saliva/buccal swab, and urine (if available) for POC testing (fingerprick blood on all participants; saliva (buccal swab)/urine only if appropriate to the test under study). Each novel POC test will be performed with strict adherence to existing biosafety precautions at each site (full PPE, performed in “red zone,” as noted) and with strict adherence to the manufacturer’s SOP. Enrolled patients receive a study number (G-####) to de-identify their laboratory specimens and clinical data. Excess fingerprick blood, saliva/buccal swab, and/or urine specimen remaining after testing with the novel POC test will be stored temporarily to allow repeat testing, if required, to resolve discordant results. Venipuncture blood (drawn in parallel with samples for POC testing) will be transported and tested by the designated reference laboratory using RT-PCR, per standard practice (below). Patients will be diagnosed based on RT-PCR results and clinical signs and symptoms by their treating physician; *results of novel POC tests will not be used for clinical management*. Basic demographic, clinical, and laboratory data will be collected for each participant (below).

*Whole Blood Testing at Centralized Laboratories:* Per standard practice, venous blood is collected and sent to a centralized testing center for RT-PCR testing per clinical routine. Prior to initiation of POC testing, the designated participating central reference laboratory will perform testing on a limited number of discarded/excess venipuncture whole blood specimens in order to confirm that performance of the novel rapid test meets expectations. Whole blood specimens sent to this laboratory for testing by RT-PCR per clinical routine will also be used to test the novel POC test in parallel. These specimens will come from multiple clinical sites and will be de-identified prior to testing on the novel POC test; patient age and sex will be recorded. Consent will not be obtained for use of these specimens, as there will be no additional procedures other than standard of care and there will be no identifiable information available to the research team. In addition, the study team will have no access to these subjects. Excess whole blood specimen remaining after testing on the novel POC test will be stored for future use in evaluations of novel diagnostics for EVD and other infectious diseases relevant to the differential diagnosis of EVD.

For consented study subjects, following testing of venous blood by RT-PCR per clinical routine, the reference laboratory will test excess venous whole blood with the novel POC assay under evaluation, as a control. Enrolled patients will have received a study number (G-####) at the time of enrollment to de-identify their laboratory samples and clinical data; after clinical testing, excess venipuncture blood sample will be de-identified, labeled with the associated study number, and tested with the novel POC assay. Depending on workflow in the reference laboratory, testing of the excess whole blood sample with the novel POC test may be performed either before, during, or after the RT-PCR testing; regardless, operators performing the novel POC assay will be blinded to the results of RT-PCR testing. If staffing and workflow allow, operators performing the RT-PCR testing will be blinded to the results of the POC test. Patients will be diagnosed based on RT-PCR results and clinical signs and symptoms by their treating physician; results of novel POC tests will not be used for clinical management. Excess whole blood specimen remaining after testing on the novel POC test will be stored for future use in evaluations of novel diagnostics for EVD and other infectious diseases relevant to the differential diagnosis of EVD.

Note: if case loads at each participating PIH clinical site are dropping rapidly (threatening study completion), we will consider testing the research samples collected an de-identified at POC (fingerstick blood, saliva/buccal swab, and/or urine) with the novel POC test in the reference lab, rather than at POC, for novel POC tests which allow sample transport. If this option is utilized, sample transport will meet all biosafety considerations applied to transport of clinical venipuncture samples.

1. **BIOSTATISTICAL ANALYSIS**
   1. Specific data variables being collected for the study

To support validation of assay performance, we will collect focused clinical and laboratory data for each participant (using designated data collection forms), to include:

Clinical Data: Age, sex, symptoms, number of days symptomatic at the time of testing, survival (for pilot discarded/excess specimen testing in the reference laboratory, only age and sex will be recorded).

Laboratory data: Ambient temperature and humidity at time of POC testing; novel POC test result [including positive/negative, valid vs invalid, reason for invalid, Ct value (for molecular assays), band density (for lateral flow tests); for lateral flow tests, test results will be evaluated and separately recorded by 2-3 independent readers in order to allow evaluation of inter-operator variability, and completed tests will be photographed to document results for subsequent review]; operator feedback on ease of fingerprick blood collection, test operation, and results interpretation (all feedback will be logged via a simple scoring system provided on the data collection form); RT-PCR result (including Ct value).

*Diagnostic testing.* Research personnel, trained by diagnostic manufacturer personnel or a study partner, if necessary, will collect specimens and conduct the testing procedure for the novel POC test on a daily basis. Data will be collected and recorded electronically, and shared with the PIs and approved team members via an online encrypted data sharing service for analysis and troubleshooting. RT-PCR run on venipuncture blood from each subject in a designated reference laboratory, according to current clinical protocol at the selected PIH site, will be used as the benchmark (reference standard) to validate the sensitivity and specificity of the novel POC assay. The specific PCR protocol will be determined by the clinical reference laboratory; all efforts will be made to ensure that this PCR protocol meets WHO standards as a benchmark. All subjects enrolled for testing with a given novel POC test will have their RT-PCR testing done with the same RT-PCR protocol in the same reference laboratory.

- 1. Study endpoints

The same study design will be applied to each test evaluation (substudy) performed under this protocol. Each substudy is a separate diagnostic accuracy study, with the primary objective of validating the performance of a novel rapid POC EBOV test in patients with suspected EVD presenting to PIH sites. Each substudy will utilize both capillary whole blood from fingerprick (tested at or near POC) and whole blood collected by venipuncture (tested in the reference laboratory) for our primary test validation. Additionally, we will collect saliva/buccal swab and/or urine, if appropriate to test specifications, in parallel for POC testing. Research testing procedures will follow a detailed substudy Standard Operating Procedure (SOP). Each novel POC test will be performed with strict adherence to the manufacturer’s SOP.

The clinical hypothesis is that the novel rapid POC test evaluated in each substudy will maintain clinical performance of >90% sensitivity and >95% specificity when compared to RT-PCR performed on venipuncture blood in the affiliated reference laboratory in Sierra Leone.

In each substudy, we will evaluate the performance of the selected novel rapid POC test at a small number (likely 1-3) of selected PIH clinical sites (ETU vs CCC), as appropriate to case volumes at the different PIH sites at the time. This will allow us to complete each study more efficiently and to avoid having to train personnel at all sites. The long-term goal of each assay validation is to ultimately be able to use the novel rapid POC test for near-patient testing. *However, during this study, results of novel POC tests will not be used for clinical management*.

- 1. Statistical methods

The results from the novel POC assay under evaluation will be compared with results from the chosen benchmark assay (RT-PCR). Sensitivity, specificity, and positive and negative predictive values will be calculated for the novel POC test using RT-PCR as the reference method. Invalid rates will be calculated, and reasons for invalid results will be tabulated. Operator assessments of ease of specimen collection and test performance will also be tabulated.

All aspects of the study, including specimen collection, testing, data entry, and data analysis, will be performed independently of the manufacturer of the novel POC test under study. All de-identified clinical and laboratory data will be analyzed by Dr. Murray and Dr. Pollock’s study team, which has no financial ties/commercial interests or personal conflicts of interest related to any participating test manufacturers.

- 1. Power analysis (e.g. sample size, evaluable subjects, etc.)

Recent positivity rates (on RT-PCR testing) observed in EVD suspects presenting to PIH sites have ranged from 25-50%. The sample size assessment is based on the anticipated sensitivity and specificity of the diagnostic test. The minimum required sample sizes given below are expressed in terms of the margin of error for a 95% confidence interval around the true sensitivity and specificity of the diagnostic test. The two-sided type I error is set at 0.05.

Number of true positive (or true negative) cases required to estimate a given level of sensitivity (or specificity) for a given novel POC test, with a selected margin of error^a^

Expected Sensitivity/Specificity       Margin of Error^b^ for Associated 95% Confidence Interval

    0.02 0.05 0.10

0.75 1941 340 75

0.80 1697 306 60

0.85          1405 265 45

0.90  1066 216 35

0.95 683 162 20

^a^ Derived using the confidence interval formula for a single proportion

^b^ Defined as half of the confidence interval width.

Based on rigorous pre-selection of candidate novel POC tests for evaluation under this protocol, we expect the true sensitivity and specificity of each novel POC test to each be approximately 85-95%. We further anticipate a ratio of patients with negative vs positive benchmark RT-PCR results ranging from 1:1 to 3:1, based on current experience at PIH field sites. We plan to enroll up to 400 patients **per substudy** (i.e. per diagnostic) in order to ensure that at least 100 of those participants will be positive by benchmark RT-PCR. Conservatively assuming a 10% invalid rate (given that each substudy will represent the earliest field testing of the diagnostic under study), this will leave at least 90 subjects positive and >90 subjects negative by benchmark RT-PCR who also have valid novel POC test results for comparison. 90 “true positive” and 90 “true negative” cases will allow us to estimate sensitivity and specificity with margins of error of 5% if sensitivity/specificity are ~95%, margins of error of 6% if sensitivity/specificity are ~90%, and margins of error of 7% if sensitivity/specificity are ~85%. In the event that we are only able to enroll 50 “true positive” and 50 “true negative” cases due to dropping case loads or resource limitations, our margins of error will still be 6% (for sensitivity/specificity of ~95%), 8% (for sensitivity/specificity of ~90%), and 10% (for sensitivity/specificity of ~85%), which we consider acceptable. For each substudy, we will assess novel POC (vs RT-PCR) results after the first 50 participants have been enrolled, to ensure that invalid rates are within expected range and that sensitivity/specificity estimates appear to match anticipated results. If the test appears to be performing as anticipated, we will continue enrollment until we have enrolled at least 100 subjects positive by benchmark RT-PCR. If resources allow, and depending on case loads, we will continue enrollment up to a total of 400 subjects per substudy.

Pilot discarded/excess specimen study: Prior to initiation of enrollment of subjects for POC testing with a novel rapid test, the designated participating central reference laboratory will perform testing on up to 200 discarded/excess venipuncture whole blood specimens in order to confirm that performance of the novel rapid test meets expectations. Specifically, excess whole blood taken from specimens sent to this laboratory for testing by RT-PCR per clinical routine will be used to test the novel POC test in parallel. Depending on workflow in the reference laboratory, it may or may not be possible to select positive vs negative (by RT-PCR) specimens for testing with the novel POC test, and specimens may have to instead be tested consecutively with the novel POC test as they arrive in the laboratory; the total of 200 specimens accounts for a 3:1 ratio of negative to positive RT-PCR results. The goal will be to test at least 50 RT-PCR+ specimens, and preferably 100 RT-PCR+ specimens, with the novel POC test. Operators of the novel POC test will be blinded to RT-PCR results. If staffing and workflow allow, operators performing the RT-PCR testing will be blinded to the results of the POC test. These specimens will come from multiple clinical sites and will be de-identified prior to testing on the novel POC test. Sensitivity and specificity of >90%/90%, respectively, will be expected in order to continue on to the POC testing phase; passage into POC testing for novel tests demonstrating sensitivity/specificity of >85%/85% will be considered. NOTE: if comparable and sufficient discarded specimen work to confirm assay performance has already been performed for the novel POC test by other investigators working in parallel in Sierra Leone, we will not repeat this testing, but rather will proceed directly to the POC testing portion of the study in order to conserve resources and time. Similarly, if case loads are dropping precipitously such that performance of a separate pilot discarded specimen study would delay the POC study to the point that the POC study would no longer be feasible, we will proceed directly to the POC study and bypass the discarded specimen study, given that we anticipate that other investigators will be performing similar discarded specimen studies for the tests under evaluation (though potentially with different reference methods). As noted, our POC study procedures also include testing excess clinical whole blood with the POC method in the reference lab as a control.

1. **RISKS AND DISCOMFORTS**
   1. Complications of surgical and non-surgical procedures, etc.

Obtaining a venous whole blood specimen (10 mL for adults, 4 mL for children) for testing by RT-PCR to determine if a person is infected with EBOV is currently standard of care. No additional blood samples will be drawn via venipuncture for study purposes. In parallel with venipuncture, an additional blood sample will be collected via fingerprick. Risks of blood drawing are typically minor, with a bruise at the puncture site being the most common risk. There is a very small risk of infection. Saliva (buccal swab) and/or urine specimens will also be collected if appropriate to the novel POC test; collection of these sample types is non-invasive. The person collecting study samples and performing the novel POC test will be wearing personal protective equipment (PPE) identical to that used for venipuncture to collect whole blood for clinical purposes. Fingerpricks will be performed with safety lancets (spring-loaded). All healthcare workers and study personnel participating in the research will be required to use personal protective equipment as directed by the standard operating procedures at each participating facility.

**The risk to phlebotomy/research teams will be minimized by the use of personal protective equipment as per Standard Operating Procedures.**

- 1. Drug side effects and toxicities

Participation in this study will not increase participants’ exposure to drug side effects or toxicities.

- 1. Device complications/malfunctions

Participation in this study will not increase participants’ exposure to device complications or malfunctions; the novel POC devices do not touch the patient, and results of novel POC tests will not be used for clinical management.

Psychosocial (non-medical) risks

The primary non-medical risk is a breach in confidentiality, resulting in the disclosure of patient information. Careful attention will be paid to maintaining confidentiality of all paper documents, electronic data, and biological specimens. Participants will each be assigned a unique ID code, which will be linked to their name in either a hard copy log stored in a locked cabinet or a password-protected form stored on an encrypted study computer. This code will be used in lieu of participants’ names on all hard copy forms, electronic datasets and biological specimens. Any paper records will be stored in a locked cabinet, and all electronic data will be password protected.

This project will observe all of the privacy and security requirements outlined by the Health Insurance Portability and Accountability Act (HIPAA) of 1996. All personally identifiable data for this study will be maintained and stored only at the participating PIH Sierra Leone sites, and study personnel will be responsible for ensuring the personal de-identification of core data needed for carrying out the study. Specifically, for all study subjects at the PIH sites, our data collection process is outlined as follows:

- 1. Verbal consent is obtained by study personnel. An informed consent log will document study subjects who verbally consent to enroll in the study.
  2. A study identification number is assigned to the enrolled subject. This ID number will be used in lieu of subjects’ names on all study materials. The key to this ID number will be stored in a locked cabinet or in a password-protected file on an encrypted computer, only accessible to study staff.
  3. Research fingerprick blood, saliva/buccal swab, and/or urine samples are collected by designated trained health care workers, labeled with their previously assigned study identification number, and tested with the novel POC test per SOP. Any images of resulted tests (e.g. lateral flow tests) will be marked only with study ID number. Data input for Instrument-based diagnostics will include study ID number only (no patient identifiers).
  4. Venipuncture blood collected for clinical testing (RT-PCR) in the reference laboratory will be labeled with personal identifiers per clinical routine. The tube will also be labeled with the assigned study identification number for later use for de-identification of excess whole blood in the reference laboratory (for testing with the novel POC test in the reference laboratory). Study staff will follow up on the clinical RT-PCR results that are returned to the PIH site per clinical routine. Ct values and results of POC tests performed in the reference lab will be collated by the reference lab and provided to study staff.
  5. Clinical and laboratory data (latter including results of POC testing with the novel test under study) are recorded by study personnel using separate clinical and laboratory data forms with no personally identifiable information; forms are labeled only with study identification number. Data will be subsequently entered into a database which similarly excludes all personally identifiable information. Copies or photographs will be made of the clinical and laboratory results forms in order to preserve this data and allow database data confirmation to be performed at a later time by study personnel.

Personally identifiable information will not be collected at any level for those subjects choosing not to participate in the study. All personally identifiable data volunteered by participants will be disassociated with the core response data prior to entry into the database. Publications or reports using the data collected in this study will only be reported or published in aggregate form with generalized results. This data collection process complies with HIPAA 45 CFR part 46.

- 1. Radiation risks

Participation in this study will not increase participants’ exposure to radiation.

1. **POTENTIAL BENEFITS**
   1. Potential benefits to participating individuals

There will be no direct benefit to subjects from this research. All study personnel will be made aware that the novel POC tests under study are for research purposes only and cannot be used to determine whether or not to initiate treatment, nor for any other clinical management decisions. There will be no payment for participation in this research study. There will be no costs to the subject for participating in this research study.

- 1. Potential benefits to society

As discussed above, development of EBOV POC tests will improve treatment of EBOV disease, facilitate studies to understand its prevalence and natural history, and ultimately lead to effective vaccines and therapies.

1. **MONITORING AND QUALITY ASSURANCE**
   1. Independent monitoring of source data

Clinical site coordinators will maintain Study Site Binders that include the Informed Consent script to be read to the patients, the approved Study Protocol, copies of the IRB and Ethics Committees approvals, and copies of the medical investigators’ CVs & Licenses. The study site coordinators and on-site medical investigators conduct regular study reviews following the Site Monitoring Protocol, when possible. During these reviews Consent and Enrollment are audited and logged and any Adverse Events or Protocol Deviations are investigated and recorded.

Data collected at the study site includes completed paper forms and electronic text and images (including automated POC test laboratory data). Study samples remaining after testing will be stored for repeat testing (if required) to resolve discordant results, and for future use in evaluations of novel diagnostics for EVD and other infectious diseases relevant to the differential diagnosis of EVD. All paper forms and electronic images will be maintained by study personnel. The limited data maintained at Harvard will include the digital database, copies of the de-identified primary data collection forms, and primary laboratory data for the novel POC tests (for platforms which store data). The database will be transmitted to the Harvard location electronically using a secure file sharing application. Study data will be maintained by study personnel on password-protected computers and transmitted to the Harvard site using password-protected, encrypted computers. Deidentified laboratory data (for automated POC tests) may also be transmitted securely via cloud-based technology for analysis by study personnel or the test manufacturer. The database, any scanned electronic forms, and raw laboratory data will be backed up regularly using password-protected, encrypted external hard drives. All host computers for study personnel with access to the study data will be password-protected and equipped with approved firewalls and antivirus software. Compliance to these data security safeguards will be ensured by requiring all project personnel to complete the NIH Computer Security Awareness Training Course or equivalent. Novel POC test manufacturers will be provided with copies of deidentified study data for product development purposes.

Samples will be logged and sequentially numbered at each site. The consent log/study ID key will be the only documentation containing patient names and other confidential information. This document will either be stored in a locked cabinet (if in hard copy) or will be password-protected and stored in an encrypted computer that will be kept in Sierra Leone. Samples and data will be identified by site and sequential number.

All data generated in this study, absent any personally identifiable information, may be sent to outside collaborators for further analysis. At the end of the study, all data and study forms will be stored for a minimum of five years at a secure location.

- 1. Safety monitoring

This study does not require a DSMB.

- 1. Outcomes monitoring

As results of novel POC tests will not be used for clinical management, this study will have no impact on clinical outcomes.

- 1. Adverse event reporting guidelines

We do not anticipate any adverse events to occur as a result of participation in this study. We will comply with the terms outlined in the PHRC guidelines on reporting unanticipated problems and adverse events. Unexpected events or occurrences will be immediately reported to the Principal Investigator, who will immediately notify PHRC and the local Research Ethics Committees. All unanticipated problems and adverse events will be reported within 7 calendar days of the date the principal investigator first becomes aware of the problem.

1. REFERENCES

1. World Health Organization. Ebola Response Roadmap Situation Report. (Accessed Jan 7, 2015; <http://www.who.int/csr/disease/ebola/situation-reports/en/)>.

2. McCarthy M. Ebola is diagnosed in traveler to US. . BMJ 2014;349:g5980.

3. Baize S, Pannetier D, Oestereich L, et al. Emergence of Zaire Ebola virus disease in Guinea. The New England journal of medicine 2014;371:1418-25.

4. Bausch DG, Schwarz L. Outbreak of ebola virus disease in Guinea: where ecology meets economy. PLoS neglected tropical diseases 2014;8:e3056.

5. Gire SK, Goba A, Andersen KG, et al. Genomic surveillance elucidates Ebola virus origin and transmission during the 2014 outbreak. Science 2014;345:1369-72.

6. Fowler RA, Fletcher T, Fischer WA, 2nd, et al. Caring for critically ill patients with ebola virus disease. Perspectives from West Africa. American journal of respiratory and critical care medicine 2014;190:733-7.

7. Kortepeter MG, Bausch DG, Bray M. Basic clinical and laboratory features of filoviral hemorrhagic fever. The Journal of infectious diseases 2011;204 Suppl 3:S810-6.

8. Kuhn JH, Dodd LE, Wahl-Jensen V, Radoshitzky SR, Bavari S, Jahrling PB. Evaluation of perceived threat differences posed by filovirus variants. Biosecurity and bioterrorism : biodefense strategy, practice, and science 2011;9:361-71.

9. Ndambi R, Akamituna P, Bonnet MJ, Tukadila AM, Muyembe-Tamfum JJ, Colebunders R. Epidemiologic and clinical aspects of the Ebola virus epidemic in Mosango, Democratic Republic of the Congo, 1995. The Journal of infectious diseases 1999;179 Suppl 1:S8-10.

10. Zaki SR, Goldsmith CS. Pathologic features of filovirus infections in humans. Current topics in microbiology and immunology 1999;235:97-116.

11. Trombley AR, Wachter L, Garrison J, et al. Comprehensive panel of real-time TaqMan polymerase chain reaction assays for detection and absolute quantification of filoviruses, arenaviruses, and New World hantaviruses. The American journal of tropical medicine and hygiene 2010;82:954-60.
